# Wavelength Calibrations in the Far Infrared (30 to 1000 Microns)

**DOI:** 10.6028/jres.067A.038

**Published:** 1963-08-01

**Authors:** K. Narahari Rao, R. V. de Vore, Earle K. Plyler

## Abstract

A discussion is presented of certain calibration procedures employed in the region 30 to 1000 microns. Calculated positions for the pure rotational absorption lines of the CO, HCN, and N_2_O molecules are given, and a map of the pure rotational absorption lines of the H_2_O molecule as recorded with a Perkin-Elmer model 301 spectrophotometer is shown.

## 1. Introduction

During the recent meetings of the Triple Commission for Spectroscopy, there have been discussions about the need for systematizing the wavelength standards and the wavelength calibration techniques for the infrared region. To partially meet this need, a report [1]^1^ has been compiled consisting of maps and the spectral positions of several vibration rotation bands occurring in the region 2.5 to 15*μ*. Evidently, it is important to provide such data for use in the far infrared at wavelengths longer than 15*μ* and extending into the millimeter region of the electromagnetic spectrum. Furthermore, it is of interest to examine critically all the currently available wavelength calibration techniques because of the impossibility of devising one procedure that can be adopted for the entire spectral region between 30 to 1000*μ*.

## 2. Experimental Procedure

**Figure 1:**
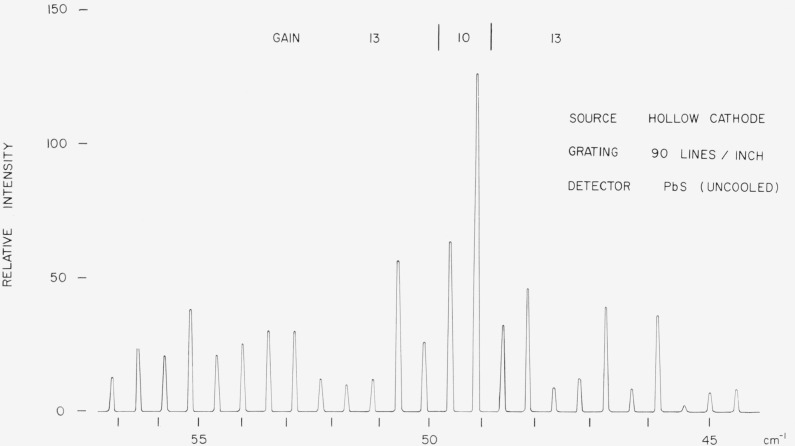
2μ *He* line recorded in higher orders (gain refers to the model 107 Perkin–Elmer amplifier).

In the far infrared region we observe mostly the pure rotational spectra of molecules. It is possible to calculate the positions of the pure rotational lines of a diatomic molecule like CO and linear triatomic molecules like HCN and N_2_O by employing the rotational constants derived from studies of the microwave and near infrared absorption spectra of these molecules. One may conceive of three different ways in which the pure rotational lines of simple molecules such as CO, N_2_O, and HCN can be used for the calibration of far infrared grating spectrometers: (i) In the case of spectrometers facilitated to determine the angular rotation of the grating, it is possible to employ the grating equation

v=nKcosecθ,

*n* being the spectral order, *K* the grating constant (expressed in cm^−1^), and *θ* the angle between the central image and the spectral line located at *v* cm^−1^. Observations on the pure rotational lines of CO, HCN, and N_2_O can lead to a value for the grating constant *K*. This value for *K* can then be used in the subsequent measurements of the far infrared lines. Evidently, we should assume that the value for *K* does not change between the time the standards (viz, CO, HCN, and N_2_O) are recorded and the time when the angular positions of the far infrared lines are measured. If several repetitive records are obtained, it is possible to minimize the errors arising from changes in the grating constant. The accuracy of the measurements made with this method depends on the accuracy with which the angular rotations of the grating can be determined. (ii) In commercial instruments, like the Perkin-Elmer model 301 far infrared spectrophotometer, the recorded data are considered to be linear in wave number units. This is accomplished by using [2]a “cosecant drive” for rotating the grating. “Pip” marks are recorded periodically on the chart. It has been our experience that, for a particular installation of the grating, the locations of these marks with respect to far infrared spectra are reproducible to accuracies of about ±0.03 cm^−1^ at 100*μ*. With these limitations in mind, it should be possible to calibrate these “pip” marks by making observations on the pure rotational lines of the above simple molecules. (iii) A technique which has been extensively employed in the near infrared spectral regions consists in the simultaneous observation, by use of double-pen recorders, of two beams of radiation passing through the spectrometer at the same time [3, 4]. One of two beams consists of the infrared spectra to be measured and the other provides a wave number scale to enable the determination of relative positions of the infrared spectral lines. If it becomes possible to impress a wave number scale on the far infrared spectra, the absolute positions of the scale can be determined by making use of the pure rotational lines of CO, N_2_O, and HCN molecules. This technique of employing two beams of radiation may prove especially useful when investigations are made with grating spectrometers operating in a vacuum. The calibrating radiation may consist of higher orders of atomic lines or higher orders of vibration rotation bands of simple molecules. Some of the atomic lines of mercury, helium and neon have sufficient intensity so that they can be detected in orders above one hundred. Figure 1 shows a part of the higher orders of the 2*μ* line of helium *ν*_air_=4858.874 cm^−1^ and *ν*_vac_=4857.525 cm^−1^ from the 85th to 110th orders as observed with a 90 lines per inch grating and a lead sulfide cell as detector. The slits of the spectrometer should be sufficiently narrow to resolve the spectra of the various orders shown in figure 1. We are emphasizing this point because the physical slits used in a far infrared spectrometer are usually quite wide (of the order of a few mm) and therefore it may become necessary to use an independent set of slits for producing wave number markers like those shown in figure 1.

This method of calibration can be checked on an instrument by using the higher orders of a definite atomic line for calibration and then measuring the positions of the higher orders of other known atomic lines.

The feasibility of employing integral relation between wavelengths of overlapping orders for obtaining spectral positions in the near infrared when modern gratings are used has been discussed adequately in previous publications [5, 6]. Since the procedures employed for ruling far infrared gratings may differ from the precise techniques used for ruling near infrared gratings, it is important to ascertain the validity of the applicability of integral relation between overlapping orders when gratings ruled for use in far infrared are employed.

**Figure 2:**
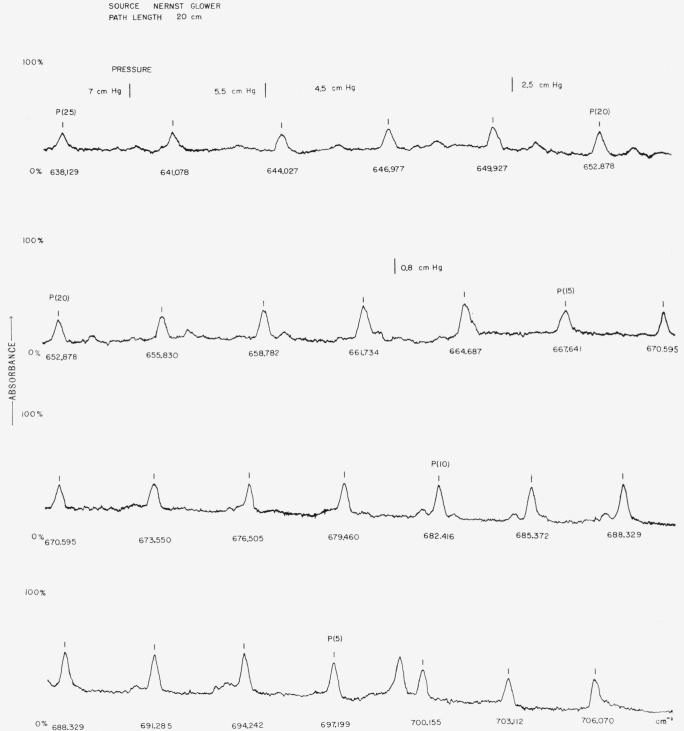

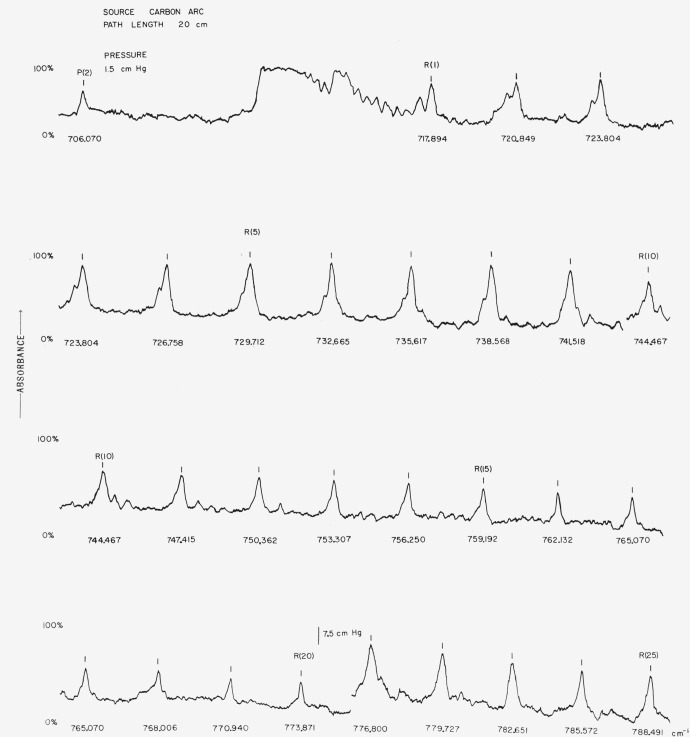
v_2_ band of *HCN* at *14*μ.

For the region from 200 to 400 cm^−1^ the pure rotational absorption spectrum of diatomic and simple polyatomic molecules are usually of low intensity. However, the pure rotational lines of water vapor are of sufficient intensity for this region. The higher orders of molecular bands can be obtained in this region and are useful. For instance, the *ν*_2_ absorption band of HCN is well suited for this purpose since it has been precisely measured [7] and the band contains many rotational lines separated by about 3 cm^−1^. Since a well resolved spectrum of this band has not been published, a trace of the observed band is included in figure 2.

## 3. Wave Numbers of the Pure Rotational Spectrum of CO, HCN, and N_2_O

Employing the rotational constants of the carbon monoxide [8, 9], hydrogen cyanide [10, 7], and the nitrous oxide [10, 11] molecules, the positions of their pure rotational spectra have been computed independently at The Ohio State University and the Bureau of Standards. The results are summarized in table 1. The path lengths and pressures necessary for the observation of these far infrared spectra are furnished as footnotes to the table.

## 4. Wave Numbers of the H_2_O Lines in the Far Infrared

**Figure 3:**
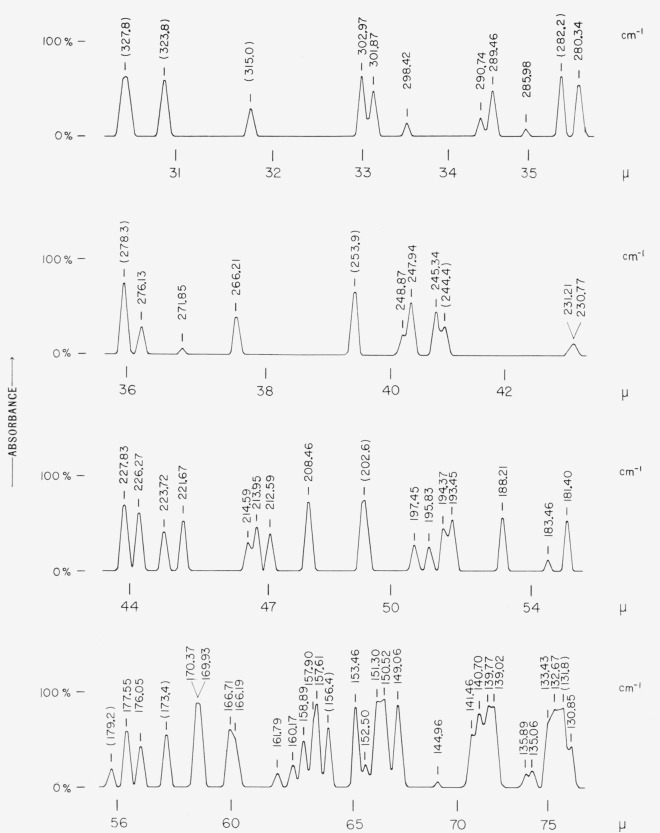

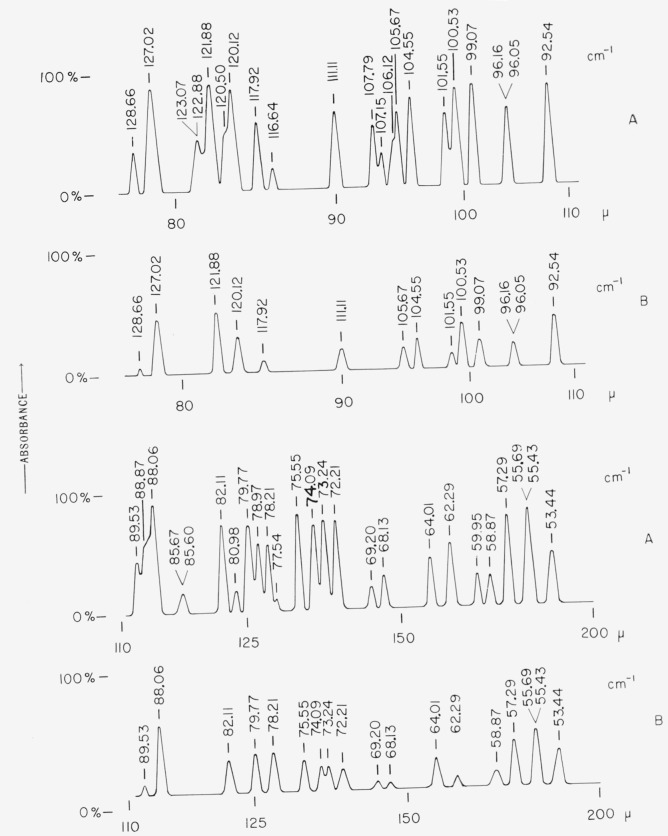
Pure rotational lines of *H_2_O* recorded with a Perkin–Elmer model 301 spectrophotometer. (A) Spectrometer flushed with dry nitrogen. (B) Spectrometer flushed with dry nitrogen and P_2_O_5_ trays were kept inside the spectrometer for 12 hours before observations were recorded. The wave number value given for each pure rotational line of the H_2_O molecule refers to vacuum. The spectrum shown above has been recorded by keeping the grating in air. It is believed that these water vapor lines may serve to identify the spectral region and provide calibrations to an accuracy of ±0.10 cm^−1^.

The pure rotational spectrum of water vapor extends from 10 to 5000*μ*, and many of the lines are intense from 25 to 400*μ*. By selecting lines which are not seriously overlapped, a number of calibrating points can be obtained. It was considered desirable to provide a map of the pure rotational lines of the H_2_O molecule with the wave numbers marked on the spectrum. Figure 3 shows such a map of the water vapor lines as obtained with a Perkin-Elmer model 301 far infrared spectrophotometer under the conditions stated in the legend for the figure. The spectral position of each of the lines is indicated on the figure. These values have been derived by Benedict [12] after analyzing all the available data pertaining to the vibration rotation and pure rotational spectra of the H_2_O molecule. The internal consistency of these data have been examined on the spectra shown in figure 3. Table 2 summarizes the findings. Tracings of the spectrum of atmospheric water vapor obtained with a small grating spectrometer in the region 600 to 166 cm^−1^ have been published by Plyler et al. [13]. Also, the paper by Rao et al. [14] gives a map and measurements (to an accuracy of ±0.02 cm^−1^) of the pure rotational lines of the H_2_O molecule, in the region 550 to 270 cm^−1^ as obtained with a 1000 lines per inch Bausch and Lomb plane replica grating installed in a Pfund-type vacuum spectrometer.

## Figures and Tables

**Table 1:** Calculated positions of the pure rotational lines* of the *C*^12^*O*^16^, *N*_2_^14^*O*^16^, and *HC*^12^*N*^14^ molecules (vacuum cm^−1^)

*J*	C^12^O^16^	N_2_^14^O^16^	HC^12^N^14^
0	3.84_5_	0.83_8_	2.95_6_
1	7.69_0_	1.67_6_	5.91_3_
2	11.53_4_	2.51_4_	8.86_9_
3	15.37_9_	3.35_2_	11.82_5_
4	19.22_2_	4.19_0_	14.78_1_
5	23.06_5_	5.02_8_	17.73_6_
6	26.90_7_	5.86_6_	20.69_1_
7	30.74_8_	6.70_4_	23.64_6_
8	34.58_8_	7.54_2_	26.59_9_
9	38.42_6_	8.38_0_	29.55_3_
10	42.26_3_	9.21_7_	32.50_5_
11	46.09_8_	10.05_5_	35.45_7_
12	49.93_2_	10.89_3_	38.40_8_
13	53.76_3_	11.73_0_	41.35_8_
14	57.59_3_	12.56_8_	44.30_7_
15	61.42_0_	13.40_5_	47.25_5_
16	65.24_5_	14.24_3_	50.20_2_
17	69.06_8_	15.08_0_	53.14_8_
18	72.88_8_	15.91_8_	56.09_2_
19	76.70_5_	16.75_5_	59.03_6_
20	80.51_9_	17.59_2_	61.97_7_
21	84.33_0_	18.42_9_	64.91_8_
22	88.13_8_	19.26_6_	67.85_6_
23	91.94_3_	20.10_3_	70.79_3_
24	95.74_4_	20.94_0_	73.72_9_
25	99.54_1_	21.77_6_	76.66_3_
26	103.33_4_	22.61_3_	79.59_5_
27	107.12_4_	23.44_9_	82.52_4_
28	110.90_9_	24.28_5_	85.45_3_
29	114.69_0_	25.12_2_	88.37_9_
30	118.46_7_	25.95_8_	91.30
31		26.79_4_	94.22
32		27.62_9_	97.14
33		28.46_5_	100.06
34		29.30_1_	102.98
35		30.13_6_	105.89
36		30.97_1_	108.80
37		31.80_6_	111.71
38		32.64_1_	114.61
39		33.47_6_	117.51
40		34.31_0_	120.41
41		35.14_5_	
42		35.97_9_	
43		36.81_3_	
44		37.64_7_	
45		38.48_1_	
46		39.31_4_	
47		40.14_7_	
48		40.98_0_	
49		41.81_3_	
50		42.64_6_	

* Path length 40 cm at pressures 2–3 cm of Hg for HCN and 40–60 cm of Hg for CO and N_2_O.

**Table 2:** Verification of the internal consistency pertaining to the data of the water vapor lines

Lines used	Interpolated* less value in column 1	Lines used	Interpolated* less value in column 3
*cm*^−1^	*cm*^−1^	*cm*^−1^	*cm*^−1^
301.87		………	………
298.42	+0.08	166.71	
290.74	+0.09	161.79	−0.03
289.46	−0.13	160.17	−0.03
285.98	+0.01	………	………
………	………		
248.87		152.50	
247.94	−0.16	151.30	−0.24
245.34	+0.13	159.52	+0.08
………	………	149.06	+0.15
		144.96	+0.13
227.83		141.46	+0.03
226.27	+0.10	140.70	−0.01
223.72	−0.06	………	………
221.67	+0.06	139.02	
214.59	+0.06	135.89	−0.06
213.95	−0.03	135.06	+0.08
212.59	−0.01	133.43	−0.14
208.46	+0.02	………	………
………	………	127.02	
194.37		122.88	−0.06
193.45	+0.03	121.88	−0.07
188.21	−0.16	120.50	+0.08
183.46	+0.08	120.12	−0.10
181.40	−0.04	117.92	+0.07
116.64	−0.07	………	………
111.11	+0.01		
107.79	0.00		
107.15	−0.01		
106.12	−0.03		
105.67	+0.04	58.87	
		57.29	+0.06
………	………	55.69	−0.04
100.53			
99.07	−0.03		
96.05	+0.07		
92.54	−0.05		
89.53	+0.09		
88.87	−0.03		
88.06	−0.09		
85.60	+0.10		
82.11	−0.01		
80.98	+0.05		
79.77	0.00		
78.97	−0.08		
78.21	+0.08		
………	………		
74.09			
73.24	+0.04		
72.21	−0.05		
………	………		

* Interpolations are made between alternate lines, e.g., the value for line 298.42 cm^−1^ was obtained by linearly interpolating between lines 301.87 and 290.74 cm^−1^.

## Footnotes

1 Figures in brackets indicate the literature references at the end of this paper.
